# Reduction in resource use with the misoprostol vaginal insert vs the dinoprostone vaginal insert for labour induction: a model-based analysis from a United Kingdom healthcare perspective

**DOI:** 10.1186/s12913-016-1278-9

**Published:** 2016-02-10

**Authors:** T. Draycott, H. van der Nelson, C. Montouchet, L. Ruff, F. Andersson

**Affiliations:** 1 Spire Bristol Hospital, The Glen, Redland Hill, Durdham Down, Bristol, BS6 6UT UK; 2 Covance Inc., Clove Building, 4 Maguire Street, London, SE1 2NQ UK; 3 Ferring Pharmaceuticals A/S, HEOR, Kay Fiskers Plads 11, DK-2300 Copenhagen S, Denmark; 4 Center for Medical Technology Assessment (CMT), Linköping University, SE-581 83 Linköping, Sweden

**Keywords:** Misoprostol, Dinoprostone, Labour induction, Resource, Vaginal insert

## Abstract

**Background:**

In view of the increasing pressure on the UK’s maternity units, new methods of labour induction are required to alleviate the burden on the National Health Service, while maintaining the quality of care for women during delivery. A model was developed to evaluate the resource use associated with misoprostol vaginal inserts (MVIs) and dinoprostone vaginal inserts (DVIs) for the induction of labour at term.

**Methods:**

The one-year Markov model estimated clinical outcomes in a hypothetical cohort of 1397 pregnant women (parous and nulliparous) induced with either MVI or DVI at Southmead Hospital, Bristol, UK. Efficacy and safety data were based on published and unpublished results from a phase III, double-blind, multicentre, randomised controlled trial. Resource use was modelled using data from labour induction during antenatal admission to patient discharge from Southmead Hospital. The model’s sensitivity to key parameters was explored in deterministic multi-way and scenario-based analyses.

**Results:**

Over one year, the model results indicated MVI use could lead to a reduction of 10,201 h (28.9 %) in the time to vaginal delivery, and an increase of 121 % and 52 % in the proportion of women achieving vaginal delivery at 12 and 24 h, respectively, compared with DVI use. Inducing women with the MVI could lead to a 25.2 % reduction in the number of midwife shifts spent managing labour induction and 451 fewer hospital bed days. These resource utilisation reductions may equate to a potential 27.4 % increase in birthing capacity at Southmead Hospital, when using the MVI instead of the DVI.

**Conclusions:**

Resource use, in addition to clinical considerations, should be considered when making decisions about labour induction methods. Our model analysis suggests the MVI is an effective method for labour induction, and could lead to a considerable reduction in resource use compared with the DVI, thereby alleviating the increasing burden of labour induction in UK hospitals.

## Background

Maternity services in the UK National Health Service (NHS) face considerable pressure to manage births–the birth rate in England is currently at its highest since 1971 [1, 2], and is increasing [3]. This coincides with UK NHS resources being cut; spending on maternity services decreased in half the geographical regions in England from 2012 to 2013 [2]. Over one third of senior midwives report insufficient budgets to support recommended minimum staffing levels [4]. The average annual number of births per midwife in the UK widely exceeds the recommended ratio of 29.5 to 1 [5], potentially impacting the quality and efficiency of care [2, 5].

Labour induction rates have also risen in recent decades [6]. Induction is typically recommended when pregnancy continuation is associated with maternal and fetal health risks [6–8]; in the UK, approximately one fifth of deliveries are induced due to safety concerns for the mother and/or fetus [9]. Vaginally-administered dinoprostone is recommended for cervical ripening prior to labour induction in the UK, augmented with oxytocin as required [7, 8]. Dinoprostone-based products are effective, but may require considerable resources [10]. One third of women induced using the dinoprostone vaginal insert (DVI; Propess®) deliver vaginally within 24 h of induction, with the remainder experiencing prolonged onset and duration of labour [11]. Furthermore, three quarters of inductions with the DVI require augmentation with oxytocin [11]; on average, oxytocin administration entails 14 h [12] of continuous monitoring by a healthcare professional [8].

Misoprostol tablets are sometimes used off-label to induce labour as it is thought that a shorter time to delivery (compared to dinoprostone) can be achieved. However, misoprostol tablets are not licensed for use in labour induction [13–18] and the evidence to support this method of induction is sparse [19, 20]. For example, national recommendations and guidelines mention use of misoprostol tablets, but only in the setting of a clinical trial [13, 14, 17]. Thus, while misoprostol may offer effective means of labour induction, off-label use is associated with a range of issues for healthcare providers, including inaccurate dosing due to tablet splitting [21, 22], the need to obtain informed consent [23, 24] and the potential risk of litigation [25, 26].

The misoprostol vaginal insert (MVI; Misodel™) − approved for labour induction − is a single-application, controlled-release, retrievable system that ripens the cervix and promotes uterotonic activity. In the EXPEDITE study [11], a phase III randomised controlled trial including 1358 women comparing the MVI (200 μg) to the DVI (10 mg), the MVI reduced median time to vaginal delivery by 11 h relative to the DVI. These reductions led to a statistically-significant increase in the proportion of vaginal deliveries occurring within 24 h, from 34 % to 55 % (*p* < 0.001). This increase may be clinically relevant, as longer labours are more likely to be associated with maternal infection than shorter labours [27]. Additionally, fewer women required oxytocin during delivery when induced with the MVI compared with the DVI, and − among those who needed it − administration duration was reduced by three hours [11].

The MVI was associated with an increase in intrapartum adverse events (AEs) necessitating medical attention compared with the DVI [11]. Uterine tachysystole requiring intervention occurred in 1.9 % and 0.6 % of women receiving the MVI and the DVI, respectively (unpublished observations from the clinical report of the EXPEDITE study). The MVI and the DVI were associated with similar rates of treatment-related neonatal intensive care unit (NICU) admissions (0.6 % vs 0.1 %, respectively), and caesarean sections (26.0 % vs 27.1 %, respectively; *P* = NS) [11].

Using efficacy and safety data reported in the EXPEDITE study, the objective of this study was to develop a model-based analysis to estimate and compare healthcare resource use associated with labour induction using the MVI instead of the DVI from a UK NHS hospital perspective.

## Methods

A model was developed in Microsoft® Excel® to estimate the time and resource use associated with labour induction using the MVI compared with the DVI. This was based on Southmead Hospital in Bristol, UK, using actual clinical data from this hospital collected between March 2013 and February 2014 as the basis for the model’s patient population, parity distribution and resource use. The model estimated resource utilisation during i) pre-active labour (the time from labour induction to onset of active labour), ii) active labour (the time from onset of active labour to delivery); iii) delivery (spontaneous or assisted vaginal delivery, or caesarean section); iv) inpatient stay (time from delivery to patient discharge from the hospital), using published [11] and unpublished safety and efficacy data from the EXPEDITE study. All unpublished clinical data from the EXPEDITE study used in the model were sourced from the associated clinical study report. AEs associated with labour induction were also estimated, based on treatment-related events reported in the EXPEDITE study. Ethics approval was not obtained as this is not applicable to this analysis. Permission to use non-patient-specific clinical data was provided by Southmead hospital–individual clinical records were not directly accessed as part of our analysis.

**Fig. 1:**
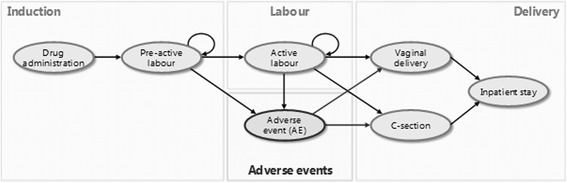
Model structure

By allowing flexibility in cycle length, a Markov structure (Fig. 1)–in which women’s outcomes and the associated costs depended on their previous state and the probability of transitioning to a different state–provided sufficient sensitivity to account for variations in the duration of labour. A cycle length of one hour was assumed to ensure sufficient accuracy in capturing differences between the efficacy and safety profiles of the two treatments.

Time-dependent probability estimates, based on unpublished Kaplan-Meier data from the EXPEDITE study, were used to model the progression of a hypothetical cohort of women induced with the DVI or the MVI through different stages of labour, as these probabilities were not estimable using median values from the published data [11]. The model assumed that different methods of labour induction were mutually exclusive and that all women entering the model received only one of the two labour induction methods. The model did not address expectant management, nor mechanical means of induction–such as the use of Foley catheters–which have been shown to be less effective in parous women than in nulliparous women [28], and are not recommended for labour induction in the UK [8]. Onset of labour and delivery were assumed to be a function of the time since induction, based on unpublished Kaplan-Meier data from the EXPEDITE study.

Other probabilities (e.g. the proportion of women requiring oxytocin and the proportion of women experiencing individual AEs) were assumed to remain constant over time. As several AEs may occur over the duration of a single labour and delivery, the model reported overall rates of treatment-related AEs rather than the proportion of women and neonates experiencing individual AEs. The incidence of AEs across nulliparous women, parous women and neonates, as reported in the EXPEDITE study, was applied to the overall cohort of women modelled. The clinically-relevant AEs (from the perspective of both mother and child) included in the model, based on EXPEDITE study data, were uterine tachysystole (with and without fetal heart rate involvement), postpartum haemorrhage, meconium-stained liquor, neonatal intensive care unit (NICU) admission, Apgar score greater than seven at five minutes, uterine rupture and neonatal acidosis [11].

Model parameters representing the number of deliveries and hospital management resource use were based on actual clinical data from the NHS Southmead Hospital in Bristol, UK, spanning one year from March 2013 to February 2014 (unpublished observations). During this one-year period, 6140 births were reported, of which 1397 (22.8 %) were induced; of the women induced, 741 (53.0 %) and 656 (47.0 %) were parous and nulliparous, respectively. Aside from these, other demographics of the model cohort were thus assumed identical to that of the population of the EXPEDITE study [11]. The model compared labour induction in a hypothetical cohort of 1397 women over one year with the DVI in the current treatment pattern, to a revised treatment pattern in which the entire cohort was instead induced using the MVI.

Model outcomes included the proportion of women requiring oxytocin and duration of oxytocin use, time to vaginal delivery, caesarean section or any delivery, number of staff shifts and bed hours, capacity for additional inductions on the ward, and frequency of AEs. Published and unpublished data from the EXPEDITE study [11] were used to model these outcomes, as shown in Table 1. The model’s sensitivity to key parameters was explored in deterministic sensitivity analyses.

**Table 1:** Model parameters informed by results from the EXPEDITE study (published [11] and unpublished data)

Model parameter	DVI (*n* = 680)	MVI (*n* = 678)
Clinical efficacy (published [11] and unpublished observations)		
Median time to vaginal delivery per patient (hours) [11]	32.8	21.5
Mean time to vaginal delivery per patient (hours)	25.2	18.2
Proportion of women achieving vaginal delivery within 12 h (%)	10	21
Proportion of women achieving vaginal delivery within 24 h (%)	36	56
Proportion of women delivered by caesarean section due to a lack of efficacy (%)	2	1
Safety (unpublished observations)		
Total adverse events (%) [11]	2.6 %	10.0 %
Uterine tachysystole without fetal heart rate involvement (requiring treatment) (%)	0.6 %	1.9 %
Uterine tachysystole with fetal heart rate involvement (late decelerations, bradycardia, or prolonged decelerations) (%)	1.2 %	6.0 %
Postpartum haemorrhage (%)	0.1 %	0.0 %
Meconium-stained liquor (%)	0.6 %	1.2 %
NICU admission (%)	0.1 %	0.6 %
Low Apgar score (%)	0.0 %	0.1 %
Uterine rupture	0.0 %	0.1 %
Neonatal acidosis (%)	0.0 %	0.1 %

To ensure the model was representative of a UK NHS hospital perspective, parameters to model labour management patterns–including the length and frequency of vaginal examinations, vital signs monitoring and oxytocin set-up carried out by midwives during pre-active and active labour–were based on real-world clinical practice data at Southmead Hospital (unpublished observations; Table 2).

**Table 2:** Resource utilisation parameters from Southmead hospital

Model parameter	Default value
Vaginal examinations (conducted by a midwife)	
Duration of examination (minutes)	10
Frequency of examinations	
Pre-active labour	Once
Active labour (without oxytocin)	Every 4 h
Active labour (with oxytocin)	Every 3 h
Vital signs monitoring (conducted by a midwife)	
Duration of monitoring (minutes)	3
Frequency of monitoring	
Pre-active labour	Every 4 h
Active labour–first stage	Every 4 h
Active labour–second stage	Every h
Oxytocin drip (set up by two midwives)	
Time to set up oxytocin drip (minutes per midwife)	10

NICE clinical guidelines on intrapartum management [15] recommend monitoring every four hours in the first stage of labour, followed by every hour during the second stage. As the EXPEDITE study [11] did not stratify active labour by stage, to replicate this pattern we assumed that the second stage of labour stage would last no more than one hour, meaning one further examination would occur during this time

## Results

### Clinical effectiveness

**Fig. 2:**
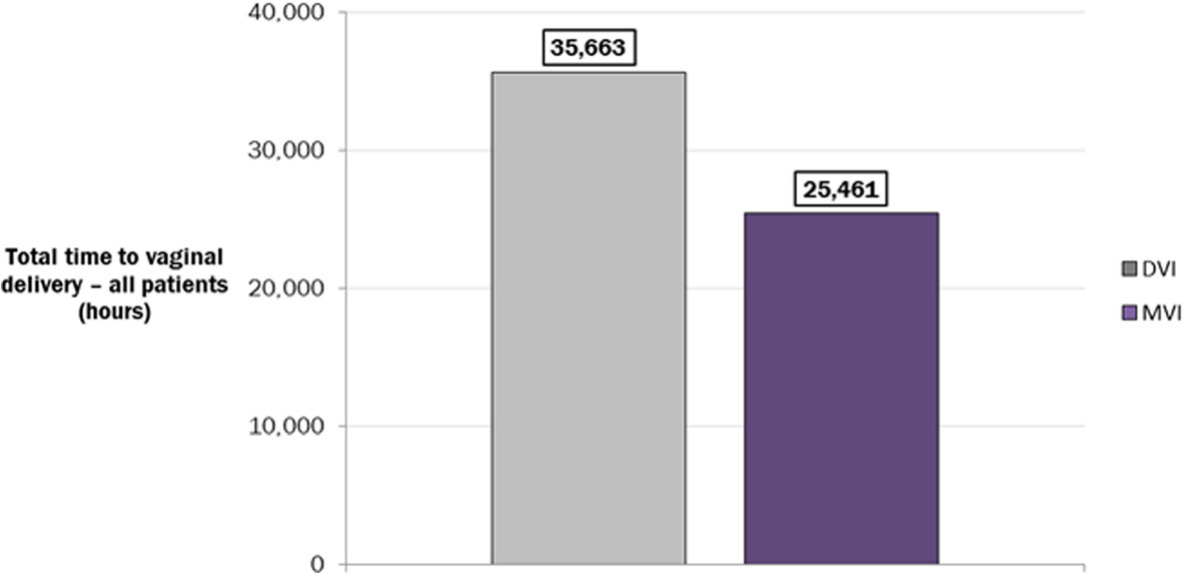
Time to vaginal delivery with the DVI vs the MVI

In the base-case analysis, the model estimated an overall 28.9 % reduction in the time to vaginal delivery (27.8 % and 30.8 % in nulliparous and parous women, respectively) when using the MVI rather than the DVI. This equated to an overall reduction of 10,201 h over one year (Fig. 2), or a reduction of 7.3 h in the time to vaginal delivery per induction (from 25.5 to 18.2 h).

**Fig. 3:**
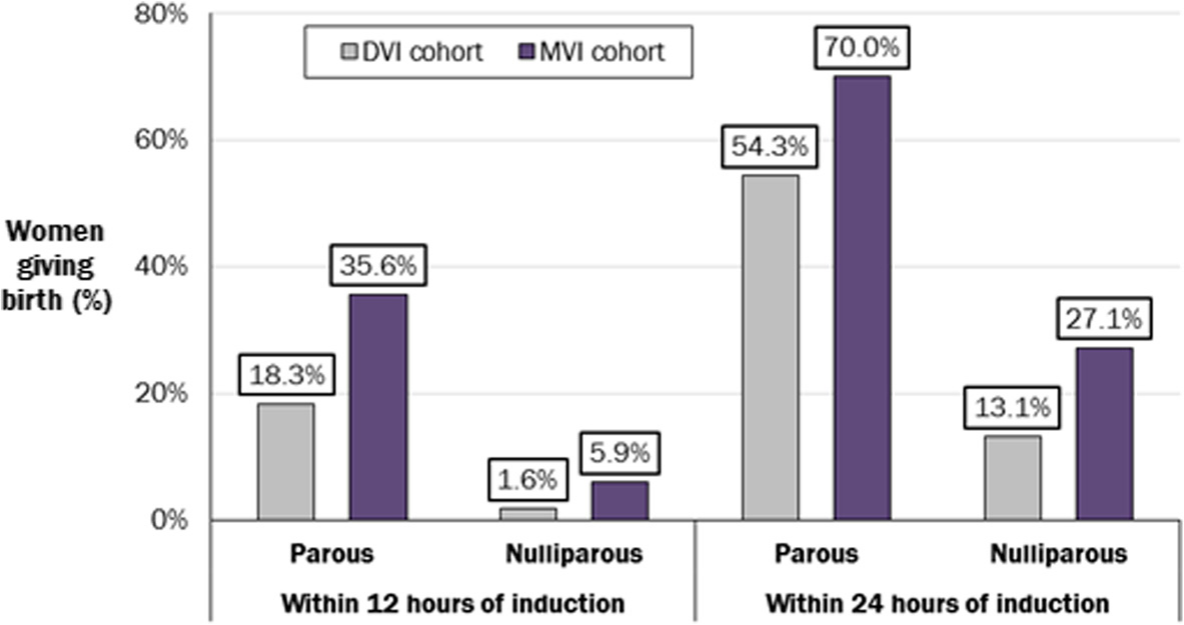
Proportion of women achieving vaginal delivery within 12 and 24 h with the DVI vs the MVI

A reduction in the proportion ofthe model cohort experiencing prolonged labour (defined as >24 h) was also estimated by the model; 68 % of women using the DVI experienced prolonged labour, compared with 53 % of women using the MVI. The proportional increase in nulliparous women delivering vaginally within 12 and 24 h was higher than in parous women: the overall proportion of women who delivered vaginally within 12 h increased 1.9-fold in parous women (from 18.3 % to 35.6 % with the DVI and the MVI, respectively) and 3.6-fold in nulliparous women (from 1.6 % to 5.9 %, respectively), whilst the proportion of women delivering vaginally within 24 h increased 1.3-fold in parous women (from 54.3 % to 70.0 %, respectively) and 2.1-fold in nulliparous women (from 13.1 % to 27.1 %, respectively) (Fig. 3).

**Fig. 4:**
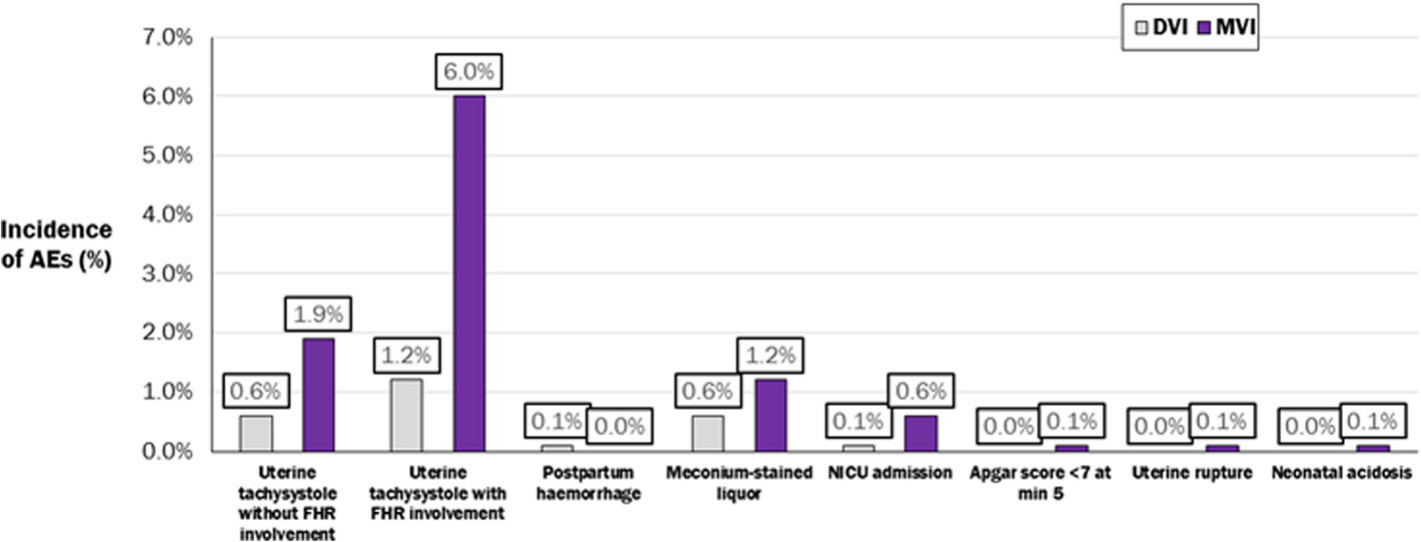
Proportion of women experiencing treatment-related AEs with the DVI vs the MVI

Use of the MVI rather than the DVI was associated with an increase in treatment-related intrapartum AEs. There were 36 AEs with the DVI compared with 140 with the MVI–an average of 0.07 additional AEs per woman. The most common treatment-related AEs were uterine tachysystole (defined as uterine activity of more than five contractions in a 10-minute window, averaged over three consecutive 10-minute periods) without fetal heart rate involvement (i.e. requiring treatment) and with fetal heart rate involvement (i.e. late decelerations, bradycardia, or prolonged decelerations), which increased 3.2-fold and 5.0-fold, respectively (Fig. 4).

Of note, a post-hoc review of AE rates reported in the EXPEDITE study was conducted by an independent, blinded expert panel, which concluded that–despite the higher incidence of certain AEs associated with the use of the MVI–neonatal outcomes were not different between the two treatment cohorts [11].

### Resource utilisation

**Fig. 5:**
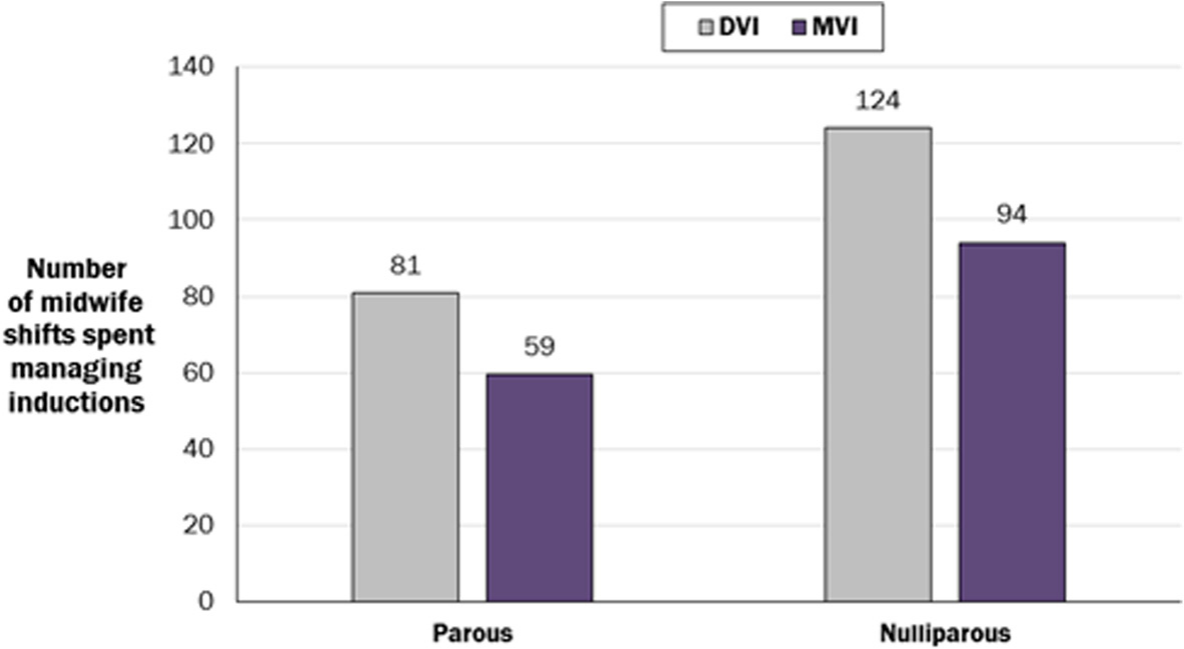
Number of midwife shifts over one year with the DVI vs the MVI

The estimated decrease in time to delivery associated with use of the MVI compared with the DVI led to decreases in hospital resource utilisation. The model estimated that 52 fewer midwife shifts were required to handle the same number of labour inductions and deliveries–a proportional reduction of 25.2 % (Fig. 5).

**Fig. 6:**
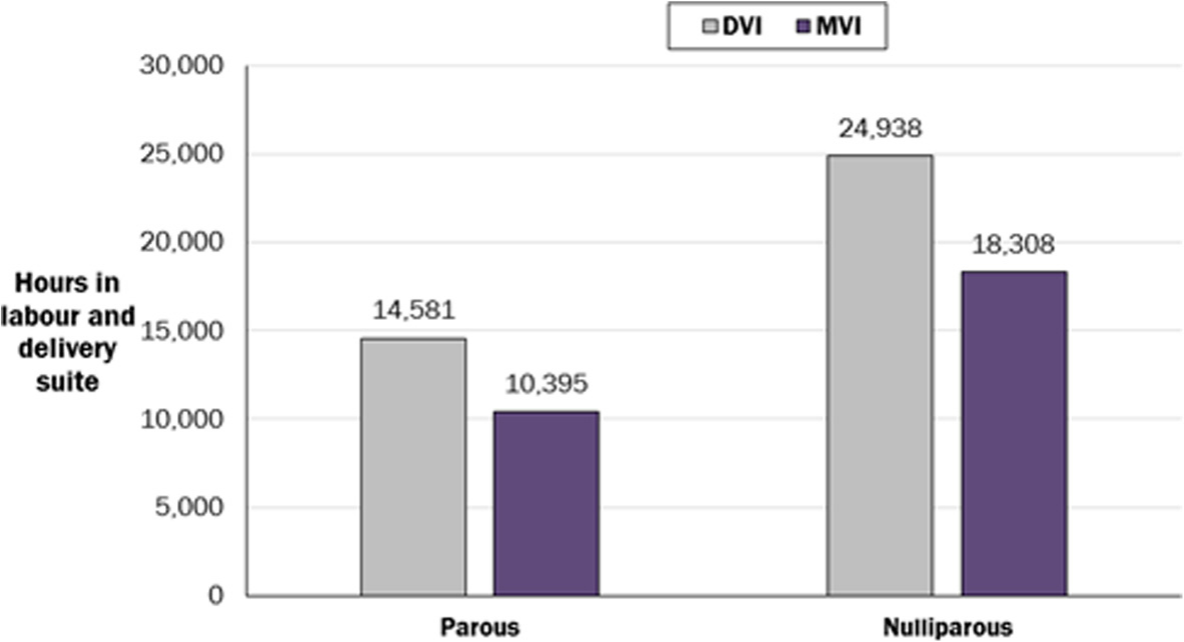
Hours of labour and delivery suite occupancy saved through the use of the MVI instead of the DVI

The model also showed that use of the MVI reduced labour and delivery suite occupancy compared with the DVI: over one year, an estimated 10,816 fewer bed hours (or 451 bed days) within the labour and delivery suite were required for women induced with the MVI rather than the DVI, a decrease of 27.4 % (Fig. 6). Specifically, 6630 and 4186 h (or 277 and 174 bed days) were saved for nulliparous and parous women, respectively. This reduction in bed hours equates to nine and six hours per nulliparous and parous woman, respectively.

In addition to decreased monitoring requirements due to shorter length of time spent in labour, part of the reduction in staff shifts was due to the decreased requirement for oxytocin to augment labour induced with the MVI. The annual number of shifts required to set up oxytocin drips was one third lower when using the MVI compared with the DVI (27 vs 43, respectively), and the number of staff shifts required for vaginal examinations and vital signs monitoring in women requiring oxytocin were halved (26 vs 53 and 6 vs 12, respectively).

As a result of these decreases in resource use associated with MVI use, the model estimated that an additional 382 inductions per year could be accommodated with the same resources if all women were induced with the MVI instead of the DVI.

### Births-per-midwife ratio

The reported births-per-midwife ratio at Southmead Hospital is 33.0 (based on clinical opinion from lead authors), with the recommended ratio at 29.5 [5]. The 27.4 % increase in capacity associated with using the MVI instead of the DVI may translate into midwives, assuming the number of labour inductions is constant, having more time to manage for each birth, leading to an estimated decrease in the births per midwife ratio from 33.0 to 24.0.

### Sensitivity analyses

**Fig. 7:**
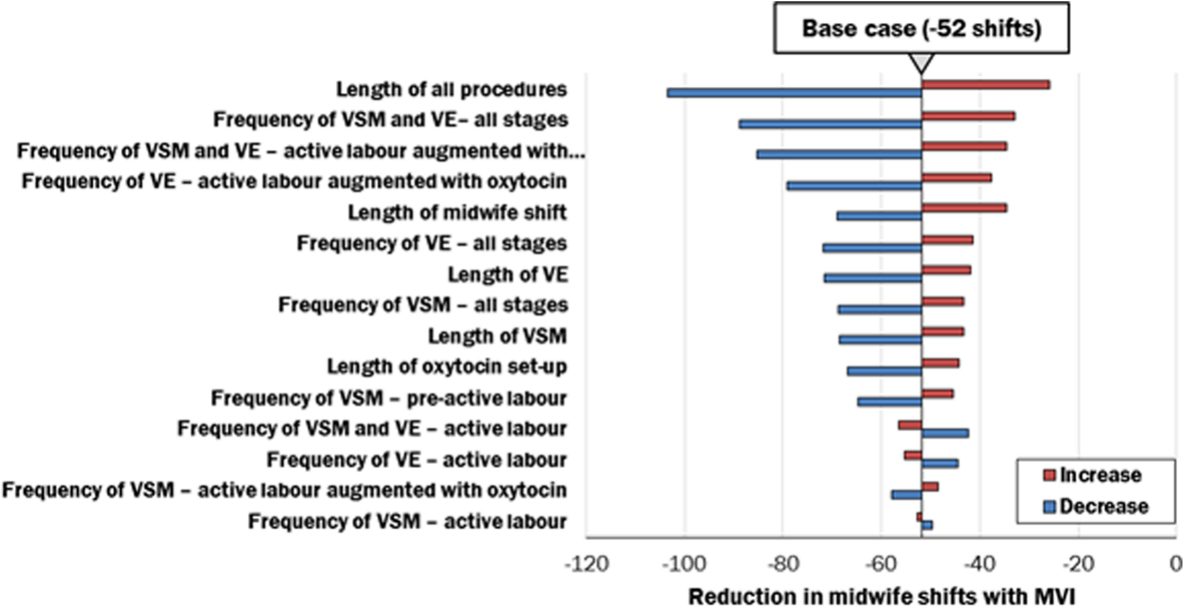
Results of sensitivity analyses. VE = vaginal examination; VSM = vital signs monitoring, Increase = doubling of parameter; decrease = halving of parameter

One-way and multi-way sensitivity analyses were conducted to test the robustness of staff capacity-focussed outcomes, by varying the following parameters by ±50 %: the duration of vital signs monitoring, vaginal examinations and oxytocin set-up, and frequency of vaginal examinations. Changing the duration of all procedures had the largest effect on the model results, but the reduction in midwife shifts due to use of the MVI instead of the DVI remained; the most conservative reduction (resulting from a scenario in which the duration of all procedures increased by 50 %) still yielded 25 fewer midwife shifts (Fig. 7).

Additionally, a scenario analysis was conducted to estimate the impact of continuous monitoring, when required, in women induced with the MVI; it was assumed that continuous monitoring would entail staff involvement equivalent to that associated with monitoring women requiring oxytocin augmentation [29]. Despite the continuous monitoring required for the MVI, decreased time to delivery led to a 7.3 % decrease in midwife shifts, or 30 fewer shifts, over one year.

## Discussion

### Main findings

Given current pressures on the NHS as a whole, and with hospitals potentially lacking sufficient resources to manage labour inductions and deliveries occurring in the UK, the potential to alleviate some of the strain and improve the efficiency and quality of care offered to women with the same resources should be a key consideration when making decisions regarding labour induction methods. Our model, based on data from real-life clinical practice and a large clinical trial, demonstrates that the MVI, by decreasing time to delivery compared with the DVI, may have a positive impact on hospital resource use and capacity. We estimate that using the MVI, rather than the current recommended standard of care, could reduce the number of midwife shifts required for labour induction and delivery, as well as the level of labour and delivery suite occupancy necessary to manage labour-induced births. This is due to the reduced time to active labour and vaginal delivery associated with the use of the MVI compared with the DVI, which in turn decreases the overall need for vital signs monitoring and vaginal examinations–in particular due to a reduction in the rate of, and duration of, oxytocin use (the administration of which requires continuous monitoring to check the progress of cervical ripening [12, 30]). A scenario analysis showed that the reduction in resource use would be maintained even if continuous monitoring were required for the MVI.

### Strengths

Though efficacy and safety data for both vaginal inserts were based on the EXPEDITE study [11] and unpublished observations, respectively, actual data from Southmead Hospital were used to inform both the demographics of the modelled cohort of women and the estimation of resource use associated with the management of labour induction in the model. This is particularly crucial in view of the expected deviation of actual practice from clinical guidelines, and supports the relevance of our model’s results to other UK hospitals.

Moreover, sensitivity analyses support the robustness of our estimates, with the results remaining largely similar throughout variation in the frequency and duration of procedures by a magnitude of 50 %–no scenario reduced midwife shift savings to fewer than 20 shifts. Given this, and despite the limitations discussed below, we expect that the decreased resource use modelled using data from Southmead Hospital would be applicable across various NHS hospitals in the UK.

### Limitations

Of course, a model-based approach is not a substitute for clinical study–our model provides numerical estimates of overall resource use based on key data from a number of sources, but as with all model-based analyses, this is a theoretical representation of what could potentially happen in actual patients. We must also acknowledge several inherent limitations to our model, which could affect the results reported here. Firstly, we do not consider the financial impact of using the MVI instead of the DVI in terms of drug costs. It must be noted, however, that drug costs represent only 2 % of total labour induction costs [31, 32], and that the majority of costs are incurred in the management of labour. Therefore, savings may be achieved through reductions in resource use. Nevertheless, we intend to conduct a follow-up analysis investigating the financial impact of using the MVI compared with the DVI. In addition, the current model assumed that the second stage of labour would last no more than one hour, despite evidence to suggest that it may last longer in nulliparous women [29]. However, as this would be true for all women, regardless of their method of induction, we did not feel this assumption would unduly favour either vaginal insert.

Another potential limitation of our model involves the use of efficacy data from the EXPEDITE trial, which was conducted in the US, to obtain UK-specific estimates. There will of course be differences between a real cohort of women in the UK and the US study population of the EXPEDITE study–such differences must be considered when forming conclusions from an theoretical analysis such as this study. This highlights two important assumptions–firstly, the similarity of the trial patient population to the general population of expectant mothers within the UK, and secondly how representative of UK clinical practice results from a US-based trial are. In light of the first assumption, while there will undoubtedly be differences between the EXPEDITE study population compared to an equivalent sample of women from the UK, the treatment arms of the EXPEDITE study were well matched, so we can assume that the differences would not differ between induction methods (as they would apply to both arms equally). Yet, we must acknowledge that there will likely be differences–data collected from 75,397 pregnancies at King’s College Hospital London (UK) [33] suggest that characteristics of expectant mothers differ between the UK and the EXPEDITE study population. This includes a higher age of mothers at birth, a higher percentage of parous pregnant women and a much lower proportion of non-white pregnant women in the UK compared to the EXPEDITE study population. Notably, the data collected at a single hospital in the UK is not necessarily fully translatable to the country-wide population, but nonetheless serves as a good estimate with which to represent a UK hospital population.

Regarding the second issue, it is well-known that clinical practice in the management of labour, delivery and AEs differs across countries, and more generally that clinical trials are not representative of real-world practice. However, certain trends (e.g. more frequent recourse to caesarean sections [34]) would not affect the resource use as estimated in our model, while parameters that would affect our model (e.g. more stringent monitoring practices) would likely apply to all women regardless of their method of induction. Moreover, the EXPEDITE study is currently the only randomised clinical trial comparing the MVI to the DVI in such a large patient population (over 1300 women), which supports the choice of this trial as the source of clinical data for our model, and also the validity of each drug’s efficacy in labour induction.

### Interpretation

The MVI is the only licensed misoprostol-based induction method to be approved for use in labour induction, avoiding any potential risks associated with off-label use. The estimated reductions we report could lead to women being discharged from hospital and returning home sooner, hypothetically increasing the birthing capacity of hospitals. In reality, this increased capacity could allow existing hospital resources to be freed–healthcare professionals may subsequently allocate their time more effectively to both induced and non-induced deliveries, especially in women receiving oxytocin (potentially facilitating more one-to-one care of these women, which is difficult to achieve in practice despite being recommended in guidelines [7, 31]). This could ultimately lead to an improvement in the quality of care received by women, as well as improved performance and efficiency of maternity services as a whole.

In line with results from the EXPEDITE study [11], the model showed a 3.9 fold increase in the overall incidence of treatment-related AEs associated with the MVI (10.0 %) compared with the DVI (2.6 %; unpublished observations from the clinical study report). The specific resource use associated with managing these AEs was not estimated in our model–while this is worth considering when estimating hospital-level resource use, methods of managing these events are likely to differ between hospitals and may fall within the care women are already receiving throughout labour induction in the hospital. However, published and unpublished results from the EXPEDITE study show that the rate of NICU admissions, caesarean sections and time to discharge after an AE with the MVI was not significantly different compared with the DVI [11], suggesting that the resource use associated with managing AEs in both cohorts would not have differed significantly. This is further supported by the similar rates of AEs resolved without sequelae, and follow-up contact due to hospital readmissions or accident and emergency visits for both MVI-induced and DVI-induced women (unpublished observations).

## Conclusion

Maternity units in the UK must manage increasing birth and labour induction rates during a period in which the NHS faces cutbacks–this has the potential to overstretch hospital resources. Resource use should therefore be integral in the decision-making process regarding labour induction methods. Our model-based analysis suggests the MVI–the first misoprostol-based preparation to be licensed for labour induction–is associated with a reduced time to, and duration of, active labour compared to the DVI, leading to an estimated reduced resource use in terms of healthcare staff time and hospital bed hours. In real-world practice, such a reduction in resource utilisation could potentially translate into improved efficiencies and optimisation of patient care, without increasing the burden on resources that hospitals already experience.

## References

[1] The Royal College of Midwives. State of maternity services report 2012. Available at: https://www.rcm.org.uk/sites/default/files/State%20of%20Maternity%20Services%20report%202012.PDF. Accessed 15 Nov 2013.

[2] The Royal College of Midwives. Maternity care cuts and staffing shortages in middle of baby boom show new figures. Available at: https://www.rcm.org.uk/content/maternity-care-cuts-and-staffing-shortages-in-middle-of-baby-boom-show-new-english-regional. Accessed 13 Nov 2013.

[3] Thomson A. UK birth rate keeps rising: Britain projected to eventually pass Germany as Europe’s most populous country. Wall Street Journal. 8 Aug 2013. Available at: http://www.wsj.com/articles/SB10001424127887323477604579000830777336474. Accessed 21 Nov 2013.

[4] Survey of senior midwives has ‘deeply worrying’ results. Midwives magazine 2011; issue 7.

[5] National Audit Office (2013). Maternity services in England.

[6] World Health Organization (2011). WHO recommendations for induction of labour.

[7] Crane J (2001). Clinical Practice Guideline Induction of labour at term. SOGC Clinical Practice Guideline.

[8] National Institute for Health and Care Excellence (2008). Induction of labour: clinical guideline.

[9] Petrou S, Taher S, Abangma G, Eddama O, Bennett P (2008). BJOG.

[10] Kalkat RK, McMillan E, Cooper H, Palmer K (2008). J Obstet Gynaecol.

[11] Wing DA, Brown R, Plante LA, Miller H, Rugarn O, Powers BL (2013). Obs Gyn.

[12] Ramsey PS, Harris DY, Ogburn PL, Heise RH, Magtibay PM, Ramin KD (2003). Am J Obstet Gynecol.

[13] Haute Autorité de Santé (HAS). Recommandations Professionnelles: Déclenchement artificiel du travail après 37 semaines d'aménorrhée. April 2008.

[14] National Institute for Health and Clinical Excellence (NICE). Clinical Guideline 70: Induction of Labour. July 2008.

[15] Arbeitsgemeinschaft der Wissenschaftlichen Medizinischen Fachgesellschaften (AWMF). Anwendung von Prostaglandinen in Geburtshilfe und Gynäkologie. August 2008.

[16] Agencia Española de Medicamentos y Productos Sanitarios. Ficha Técnica. Misofar 25 microgramos comprimidos vaginales. December 2012.

[17] SOGC Clinical Practice Guideline (2013). Induction of Labor: Review. No. 296. September 2013 (reviewed March 2015).

[18] Centro Nacional de Excelencia Technológica en Salud (2009). Guía de Práctica Clínica.

[19] Compte rendu scientifique (2007). 35èmes Assises Nationales des Sages-femmes. La Rochelle.

[20] Hofmeyr GJ, Gülmezoglu AM, Pileggi C. Cochrane Database Syst Rev 2010;(10):CD000941. 10.1002/14651858.CD000941.pub2 PMC7061246 20927722

[21] Williams MC, Tsibris JC, Davis G, Baiano J, O'Brien WF. Am J Obstet Gynecol 2002;187(3):615–9. 10.1067/mob.2002.124959 12237637

[22] Tang OS, Schweer H, Seyberth HW, Lee SW, Ho PC. Hum Reprod 2002;17(2):332–6. 10.1093/humrep/17.2.332 11821273

[23] Marshall JE (2002). Informed Consent during the Intrapartum period: An Observational Study of the Interaction between Health Professionals and Women in Labour. 26th Triennial Congress of the International Confederation of Midwives Book of Proceedings (abstract 135).

[24] Fukuda H, Imanaka Y, Kobuse H, Hayashida K, Murakami G. J Eval Clin Pract 2009;15(2):234–41. 10.1111/j.1365-2753.2008.00987.x 19335478

[25] Oden M (2009). J Perinat Educ.

[26] American Association of Legal Nurse Consultants (2004). The LiNC.

[27] Mandruzzato G, Alfirevic Z, Chervenak F, Gruenebaum A, Heimstad R, Heinonen S (2010). J Perinat Med.

[28] Jozwiak M, Bloemenkamp KW, Kelly AJ, Mol BW, Irion O, Boulvain M (2012). Cochrane Database Syst Rev.

[29] National Institute for Health and Care Excellence (2007). Intrapartum care. Care of healthy women and their babies during childbirth.

[30] Hofmeyr GJ, Gülmezoglu AM, Pileggi C (2010). Cochrane Database Syst Rev.

[31] Calder AA, Loughney AD, Weir CJ, Barber JW (2008). BJOG.

[32] HRG tariffs 2012–2013; tariff information spreadsheet. https://www.gov.uk/government/publications/confirmation-of-payment-by-results-pbr-arrangements-for-2012-13. Accessed 22 Nov 2013.

[33] Poon LCY, Volpe N, Muto B, Argyro S, Nicolaides KH (2012). Fetal Diagn Ther.

[34] Guerra GV, Cecatti JG, Souza JP, Faúndes A, Morais SS, Gülmezoglu AM (2009). BJOG.

